# Empathy, Autonomy, and Social Expectations in Surrogate Decision-Making Among Taiwanese Adults

**DOI:** 10.1093/geroni/igaf122.2612

**Published:** 2025-12-31

**Authors:** Duan-Rung Chen, Yu-Chen Juang

**Affiliations:** National Taiwan University, Taipei, Taipei, Taiwan (Republic of China); National Taiwan University, Taipei, Taipei, Taiwan (Republic of China)

## Abstract

**Purpose:**

Surrogate decision-making involves making choices for individuals unable to express their preferences. In many Asian societies, this process is family-centered, with elders—typically sons or senior members—making decisions based on hierarchy, harmony, and collective responsibility. Tunney and Ziegler’s (2015) typology categorizes surrogate decisions into four types: (1) Substituted Judgment, reflecting the patient’s prior wishes; (2) Benevolent Decision-Making, prioritizing the patient’s best interests; (3) Egocentric Decision-Making, influenced by external pressures; and (4) Projective Decision-Making, where surrogates assume patients would choose as they would. This study examines the most prevalent decision-making type in Taiwan and the influence of gender.

**Methods:**

An online questionnaire was distributed via social media, targeting a broad demographic, including older adults. Conducted from October 2024 to January 2025, it gathered 2,000 responses, with 25% aged 65 or older. Of the respondents, 10.9% worked in medical fields, 70% held a college degree, and 51% were male.

**Results:**

Gender differences emerged in surrogate decision-making. Women prioritized Substituted Judgment, Projective, and Benevolent Decision-Making, incorporating patient preferences, empathy, and minimizing suffering. Men leaned toward Egocentric Decision-Making, influenced by societal expectations and familial opinions, prioritizing external perceptions over patient well-being.

**Conclusion:**

Cultural norms and gender roles shape surrogate decisions. Women tend to be patient-centered, while men emphasize social responsibility. Understanding these differences can enhance ethical frameworks, ensuring surrogate decisions align more closely with patient autonomy and well-being.

